# Long-Term Durability of Tenofovir-Based Antiretroviral Therapy in Relation to the Co-Administration of Other Drug Classes in Routine Clinical Practice

**DOI:** 10.1371/journal.pone.0160761

**Published:** 2016-10-07

**Authors:** Silvia Costarelli, Alessandro Cozzi-Lepri, Giuseppe Lapadula, Stefano Bonora, Giordano Madeddu, Franco Maggiolo, Andrea Antinori, Massimo Galli, Giovanni Di Perri, Pierluigi Viale, Antonella d’Arminio Monforte, Andrea Gori, ICONA Foundation Study Group

**Affiliations:** 1 Department of Infectious Diseases, San Gerardo Hospital, University of Milano-Bicocca, Monza, Italy; 2 Department of Virology, Royal Free and University College Medical School, London, United Kingdom; 3 Department of Medical Sciences, University of Torino, Torino, Italy; 4 Department of Clinical and Experimental Medicine, Unit of Infectious Diseases, University of Sassari, Sassari, Italy; 5 Department of Infectious Diseases, Papa Giovanni XXIII Hospital, Bergamo, Italy; 6 National Institute for Infectious Diseases-IRCCS, Rome, Italy; 7 Department of Clinical and Biomedical Sciences “Luigi Sacco”, Section of Infectious Diseases, University of Milan, Milan, Italy; 8 Clinic of Infectious Diseases, S.Orsola-Malpighi Hospital, University of Bologna, Bologna, Italy; 9 Unit of Infectious Diseases, Department of Health Sciences, University of Milan, Milan, Italy; National and Kapodistrian University of Athens, GREECE

## Abstract

**Background:**

In clinical trials, toxicity leading to tenofovir disoproxil fumarate (TDF) discontinuation is rare (3% by 2 years); however in clinical practice it seems to be higher, particularly when TDF is co-administered with ritonavir-boosted protease inhibitors (PI/r). Aims of this study were to assess the rate of TDF discontinuations in clinical practice and to identify factors associated with the risk of stopping TDF.

**Methods:**

All antiretroviral treatment (ART)-naive patients initiating a TDF-based regimen were selected from the ICONA Foundation Study cohort. The primary outcome was TDF discontinuation regardless of the reason; secondary outcome measures were TDF discontinuation due to toxicity and selective TDF discontinuation (that is, TDF discontinuation or substitution, maintaining unchanged the remaining antiretroviral treatment).

**Results:**

3,618 ART-naïve patients were included: 54% started a PI/r-based and 46% a NNRTI-based based regimen. Two-hundred-seventy-seven patients discontinued TDF and reintroduced ART within 30 days without TDF. The probability of TDF discontinuation regardless of the reason was of 7.4% (95%CI:6.4–8.5) by 2 years and 14.1% (95%CI:12.2–16.1) by 5 years. The 5-year KM estimates in the PI/r vs. NNRTI group were 20.4% vs. 7.6%, respectively (log-rank p = 0.0001), for the outcome of stopping regardless of the reason, and 10.7% vs. 4.7% (p = 0.0001) for discontinuation due to toxicity. PI/r use and lower eGFR were associated with an increased risk of discontinuing TDF.

**Conclusion:**

In our cohort, the frequency of TDF discontinuations was higher than that observed in clinical trials. Co-administration of TDF with PI/r was associated with an increased rate of TDF discontinuations. Further studies are needed to clarify the mechanisms that might have led to this outcome.

## Background

Antiretroviral therapy (ART) regimens could be associated with a range of toxicities. Although the incidence of discontinuation because of intolerance/toxicity has declined over time, it remains the major cause of drug discontinuation.[1] Tenofovir disoproxil fumarate (TDF) is a widely prescribed nucleotide reverse transcriptase inhibitor (NRTI) for HIV-1 infection. Possible TDF adverse events include renal tubule damage, Fanconi’s syndrome, nephrogenic diabetes insipidus and osteopenia/osteoporosis. Although the incidence of renal disease can be reduced by ART,[2] current use and cumulative exposure to TDF have been associated with estimated glomerular filtration rate (eGFR) reduction and/or increased incidence of chronic kidney disease (CKD).[3–6] Moreover, cumulative TDF exposure has been associated with reduced bone mineral density and increased osteoporotic fracture risk.[7]

TDF-related renal toxicity seems to be enhanced by concurrent administration of ritonavir-boosted protease inhibitors (PI/r), particularly atazanavir/ritonavir (ATZ/r) and lopinavir/ritonavir (LPV/r).[4, 8, 9] Similarly, a steeper increase in bone resorption markers and more marked reduction in bone mineral density were observed in three randomized clinical trials, when TDF was associated with a PI/r.[10–12]

Toxicity leading to discontinuation of TDF is a rare occurrence, in clinical trials, ranging from 0 to 3% by 2 years from starting the drug.[13–18] Nonetheless, in clinical practice, the proportion of TDF discontinuations due to toxicity or side effects seems to be higher, but it remains largely unexplored.

The aim of our study was to describe the use of TDF as part of first-line ART initiated from ART-naïve in clinical practice, to assess the rate of its discontinuation over time and to explore factors associated with the risk of TDF discontinuation (with the focus on the drug class of the third drug initiated with TDF).

## Methods

Data from the Icona Foundation Study database were used. A detailed description of the cohort has been provided elsewhere.[19, 20] In brief, the ICONA Foundation Study is an Italian multicentre prospective observational cohort study of HIV-1-positive persons enrolled since 1997. This cohort consists of more than 12,000 patients, recruited in 71 infectious disease units in Italy, 41 of which still provide new enrolments and updated follow-up of the persons enrolled. Eligible patients are those who, for whatever reason, were naive to antiretroviral drugs at the time of enrolment. Demographic, pre-enrolment, clinical and laboratory data and information on the specific therapies are collected for all participants and recorded online. Reasons (up to three) for discontinuing drugs according to the treating physician are also reported on a standardized case report form. Only the main reason for discontinuation per antiretroviral drug was used in the analysis. All data are updated at the occurrence of any clinical event and, in the absence of such an event, at least every 6 months.

Patients from the ICONA Foundation Study were included in the present analysis if they had initiated a TDF-containing regimen together with a non-nucleoside reverse transcriptase inhibitors (NNRTI) or a PI/r while naïve to antiretrovirals, between January 1^st^ 2003 and June 30^th^ 2014, and they had been treated with TDF for >30 days. Patients who ever tested positive for hepatitis B surface antigen over follow-up were excluded. This was done because persistence on TDF treatment, despite toxicities, was assumed to be higher among those with hepatitis B co-infection than among HIV monoinfected patients. Follow-up accrued from the date of TDF initiation up to its discontinuation or to the last recorded clinical visit. The reasons for TDF discontinuation, as reported by the treating physicians, was used to classify the interruptions.

The primary outcome was TDF discontinuation regardless of the cause. Secondary outcome measure was TDF discontinuation due to toxicity, as reported by the treating physician. In the secondary outcome analysis, follow up was truncated at the date of last clinical visit if a person had discontinued for a reason different from toxicity. This was done because we were interested in predicting how many people stopped TDF due to toxicity. We used the marginal risk which reflects both the causal effect of covariates on the risk of stopping TDF because of toxicity but also other possible mechanisms related to the competing events (e.g., interruptions for other causes which were not relevant here). The analysis of the risk of interruption due to toxicity was further restricted to only discontinuations due to renal toxicity, again using a marginal model approach and classifying the reason for discontinuation according to the reason reported by the treating physician. Eventually, all analyses were repeated considering only selective TDF discontinuations (TDF interruption or substitution, while maintaining unchanged the remaining antiretroviral treatment) as the outcome of interest, in order to minimize the possible effect of the companion drugs’ side effects on the decision to interrupt TDF. So, this analysis is designed to evaluate the rate of treatment discontinuation truly attributable to TDF and not to other drugs. In all analyses, changes in formulation and interruptions followed by re-initiation of TDF-based regimens within 1 month and/or TDF discontinuations in the context of a complete ART discontinuation lasting >1 month did not count as events.

Survival analysis using Kaplan Meyer (KM) and Cox proportional hazards model were used. An intention-to-treat (ITT) approach, ignoring switches of PI/r, NNRTI or other nucleoside reverse transcriptase inhibitors (NRTI), was performed. Besides the drug class co-administrated with TDF (PI/r vs. NNRT), the following covariates were included in the multivariable Cox model: demographics, mode of HIV transmission, hepatitis C coinfection (defined as serum reactivity for hepatitis C virus antibody), baseline eGFR (calculated using the CKD-Epi formula) [21] and CD4+ T-cell count, diagnosis of diabetes, lipid assessment (total cholesterol and HDL cholesterol), use of blood pressure lowering drugs and statins, calendar year. These were chosen a priori as potentially associated with both the choice of the initial class of the third drug and the risk of stopping TDF. Cox regression models were stratified by clinical site.

Finally we estimated the variation of eGFR over time since TDF initiation and whether it was different according to third-drug class used (PI/r or NNRTI), using a mixed linear model with random intercept and slope. This was done to supplement the main analysis based on the reason reported by the clinician which is likely to be a less objective endpoint influenced by beliefs engrained in clinical practice.

All statistical analyses were performed using SAS 9.4 statistical software (SAS Institute Inc., Cary, NC, USA, 2014). All P-values presented are two sided and a P-value <0.05 indicated conventional statistical significance.

### Ethics Statement

Patients included in the ICONA Foundation Study provide, at enrolment, written informed consent to include their clinical data in the ICONA dataabse for scientific purposes. The data are anonymized and the database is hosted by the ICONA foundation, in compliance with current Italian regulations. The study was approved by the Ethical Committee of the Hospital “San Paolo”, Università degli Studi, Milan (Coordinating Centre) and those of the following Institutions: Università “G. D'Annunzio”, Ospedale SS. Annunziata, Chieti; Ospedale Civile Santo Spirito, Pescara; Azienda Ospedaliera “D. Cotugno”, Napoli; Azienda Ospedaliera Universitaria “Federico II”, Napoli; Policlinico “S. Orsola Malpighi”, Bologna; Università degli Studi, Arcispedale S. Maria Nuova, Reggio Emilia; Università degli Studi, Policlinico di Modena; Azienda Ospedaliera Universitaria “Arcispedale S. Anna”, Ferrara; A.O.U. “Santa Maria della Misericordia”, Udine; INMI IRCCS “Lazzaro Spallanzani”, Roma; Azienda Ospedaliera Universitaria Policlinico Tor Vergata, Roma; Centro Coordinamento AIDS, Latina (Roma); Policlinico Gemelli, Università Cattolica, Roma; Policlinico Umberto I, Università La Sapienza, Roma; Ospedale Bel Colle Viterbo, Viterbo; Università degli Studi, Ospedale San Martino, Genova; Ospedali Galliera, Genova; Ospedali Riuniti, Bergamo; Università degli Studi, Spedali Civili, Brescia;

Ospedale di Circolo, Busto Arsizio; Ospedale “A. Manzoni”, Lecco; Ospedale “Luigi Sacco”, Milano; Università degli Studi, IRCCS “San Raffaele”, Milano; Ospedale Niguarda, Milano; Ospedale “San Gerardo”, Monza; Ospedale Torrette, Ancona; Università Politecnica Marche, Ancona; Ospedale Generale Provinciale, Macerata; Università di Torino, Ospedale Amedeo di Savoia, Torino; Università degli studi, Bari; Policlinico Universitario di Monserrato, Cagliari; Università degli Studi, Sassari; Ospedale Garibaldi, Presidio Ospedaliero Nesima, Catania; Università di Messina;

Azienda Ospedaliera “Umberto I”, Siracusa; Ospedale “S.M. Annunziata”, Firenze; Azienda Ospedaliera Universitaria Senese, Siena; Università degli Studi, Policlinico Monteluce, Perugia; Azienda Ospedaliera “Santa Maria”, Terni.

## Results

### Patients’ characteristics

Three thousands six hundred and eighteen HIV-positive patients were enrolled and followed for a total of 8,043 patient-years of follow-up. One thousand six-hundred sixty-nine patients (46%) started TDF as part of a NNRTI- based regimen and the remaining 1,949 (54%) a PI/r based regimen. Their median age was 38 years-old and their median baseline eGFR was 106 ml/min. Patients on PI/r based regimen were more likely to be female (p<0.001), older (p = 0.02) and previously diagnosed with AIDS (p<0.001) and to have a lower CD4-T cell count (p<0.001) and a higher HIV viral load (p<0.001, Table 1). Among patients on PI/r based regimen, 783 (40%) were on ATZ/r, 676 (35%) were on darunavir/ritonavir (DRV/r) and 490 (25%) were on LPV/r. Patients who started TDF as a part of a integrase- inhibitor based regimen were only 8, and we decided not to include them in the analysis. A detailed description of the characteristics of the patients is shown in Table 1.

**Table 1:** Characteristics of the patients initiating a tenofovir-containing regimen, grouped by “third drug” class.

Characteristics	Third-drug class		p-value	Total
				N = 3618
	NNRTI	PI/r		
	N = 1669	N = 1949		
**Gender, n (%)**				
Female	296 (17.7%)	440 (22.6%)	<0.001	736 (20.3%)
**Mode of HIV transmission, n (%)**				
Intravenous drug use	148 (8.9%)	209 (10.8%)		357 (9.9%)
Homosexual contacts	723 (43.5%)	721 (37.1%)		1444 (40.1%)
Heterosexual contacts	667 (40.0%)	858 (44%)		1525 (42.2%)
Other/unknown	124 (7.5%)	154 (7.9%)	0.001	278 (7.7%)
**Ethnicity, n (%)**				
Black	85 (5.1%)	151 (7.7%)	0.001	236 (6.5%)
**AIDS diagnosis, n (%)**				
Yes	69 (4.1%)	146 (7.5%)	<0.001	215 (5.9%)
**NRTIs, n (%)**				
FTC	1536 (92%)	1869 (95.9%)		3405 (94.1%)
3TC	115 (6.9%)	54 (2.8%)		169 (4.7%)
Other	18 (1.1%)	26 (1.3%)	<0.001	5 (0.1%)
**HCVAb, n (%)**				
Negative	798 (47.8%)	725 (37.2%)		1523 (42.1%)
Positive	130 (7.8%)	127 (6.5%)		257 (7.1%)
Not tested	741 (44.4%)	1097 (56.3%)	0.817	1838 (50.8%)
**Age, years**				
Median (IQR)	37 (31,43)	38 (32,45)	0.020	38 (32,44)
**CD4, count, cells/mmc**				
Median (IQR)	330 (235,419)	253 (110,372)	<0.001	296 (165,397)
**CD4 count nadir, cells/mmc**				
Median (IQR)	311 (218,396)	243 (105,353)	<0.001	280 (154,378)
**CD8 count, cells/mmc**				
Median (IQR)	907 (656,1256)	824 (550,1210)	<0.001	866 (592,1233)
**Viral load, log10 copies/mL**				
Median (IQR)	4.60 (4,5.09)	4.90 (4.18,5.43)	<0.001	4.74 (4.07,5.25)
**Follow-up, months**				
Median (IQR)	19 (7,41)	19 (6,34)	0.012	19 (6,36)
**Time from enrollment to date of starting antiretroviral treatment, months**				
Median (IQR)	2 (0,18)	1 (0,4)	<0.001	1 (0,11)
**Calendar year of baseline**				
Median (IQR)	2011 (2009–2013)	2011 (2010–2012)	0.817	2011 (2009–2012)
**eGFR (CKD-epi formula), ml/min**				
Median (IQR)	105.9 (93.77,115.4)	106.5 (93.50, 116.5)	0.369	106.2 (93.62–116.2)

List of abbreviations: 3TC, lamivudine; AIDS, acquired immune deficiency syndrome; CKD-epi, chronic kidney disease epidemiology collaboration [21]; eGFR, estimated glomerular filtration rate; FTC, emtricitabine; HCVAb, hepatitis C virus antibodies; HIV, human immunodeficiency virus; IQR, interquartile range; NRTI, nucleoside reverse transcriptase inhibitor; NNRTI, non-nucleoside reverse transcriptase inhibitor; PI/r, ritonavir-boosted protease inhibitor

### Risk of tenofovir discontinuation

**Fig 1:**
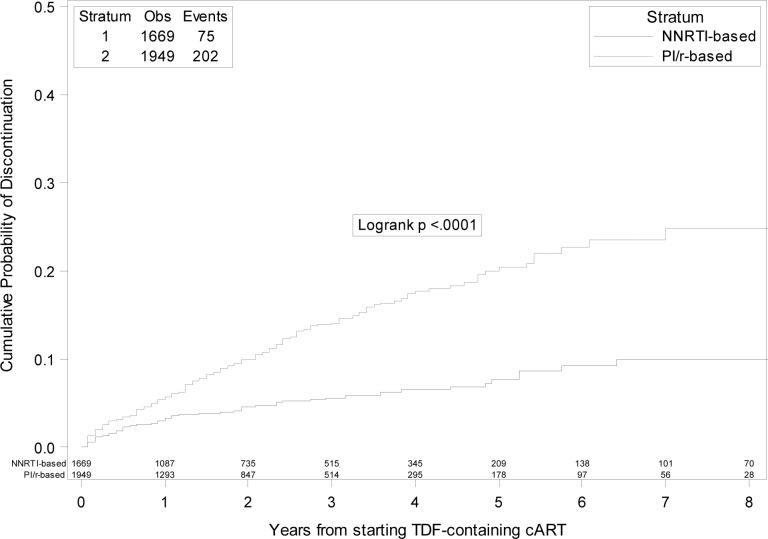
Kaplan-Meier estimates of risk of tenofovir discontinuation regardless of the reason in boosted protease inhibitors versus non-nucleoside reverse transcriptase inhibitors groups.

A total of 277 cases of TDF discontinuation were observed, of whom 202 in PI/r group and 75 in NNRTI group, respectively. The probability of discontinuation of TDF regardless of the reason was of 7.4% (95% CI: 6.4–8.5) by 2 years and 14.1% (95%CI 12.2–16.1) by 5 years. When patients were grouped according to the third-drug class started with TDF, the 5- year KM estimates of TDF discontinuation were 7.6% (95%CI: 5.5–9.7)) and 20.4% (95%CI 17.2–23.6) in the NNRTI and PI/r group, respectively (log-rank p<0.001) (Fig 1). Among the 277 patients who stopped TDF, 123 (44.4%) switched to an abacavir/lamivudine-containing regimen, 50 (18%) to a regimen containing the sole lamivudine or emtricitabine, 29 (10.5%) to a zidovudine/lamivudine-containing regimen, 2 to other NRTI-combinations and 64 (23.1%) to NRTI-sparing combinations. A detailed description of the composition of the regimen started after the discontinuation according to the initial third drug is depicted in Table 2.

**Table 2:** Composition of the NRTI back-bone of the regimen started after the discontinuation of tenofovir (rows), according to the initial third drug (columns)–All tenofovir discontinuations.

	Regimen started					
New regimen after stop of TDF	TDF/FTC or TDF/3TC					
	plus					
	Efavirenz	Nevirapine	Lopinavir	Atazanavir	Darunavir	Any drug
NRTI-sparing regimen	12	0	25	17	10	64 (23.1%)
Abacavir/lamivudine	31	5	37	27	23	123 (44.4%)
Zidovudine/lamvudine	15	1	6	5	2	29 (10.5%)
Lamivudine or emtricitabine only	4	0	5	28	13	50 (18.1%)
Didanosine ± lamivudine or emtricitabine	6	0	3	0	0	9 (3.2%)
Other NRTI combinations	1	0	0	1	0	2 (0.7%)
Total	69 (24.9%)	6 (2.2%)	76(27.4%)	78 (28.2%)	48 (17.3%)	277 (100%)

A multivariable Cox regression analysis assessing time fixed factors at baseline associated with the risk of TDF discontinuation was run (full results in Table 3A). In this model, initiation of a PI/r-based treatment was associated with a significantly higher risk of TDF discontinuation (HR 1.70; 95%CI 1.16–2.48). Moreover, a lower eGFR at ART initiation was an additional independent risk factor (per 10 ml/min decrease, HR 1.19; 95%CI 1.08–1.32). Among patients starting a PI/r-based regimen, the hazard of TDF discontinuation did not differ to a significant extent comparing the different PI/r (HR 0.99; 95%CI 0.66–1.48 and HR 1.57; 95%CI 0.9–2.73, for use of ATV/r and DRV/r *versus* LPV/r, respectively).

**Table 3:** (a,b,c) Multivariable Cox regression analysis assessing factors associated with tenofovir discontinuation regardless of the reason, because of toxicity and with selective tenofovir discontinuation.

	Crude and adjusted relative hazards			
Outcomes	Crude RH (95%CI)	p-value	Adjusted* RH (95%CI)	p-value
***(A) Discontinuation of tenofovir regardless of the reason***				
***Drug Class***				
NNRTI	1.00		1.00	
PI	2.50 (1.91–3.26)	<0.001	1.70 (1.16–2.48)	0.006
***Baseline weight*, *Kg***				
per 10 heavier	0.94 (0.84–1.05)	0.263	0.95 (0.81–1.11)	0.534
***Baseline eGFR*, *ml/min***				
per 10 lower	1.18 (1.10–1.27)	<0.001	1.19 (1.08–1.32)	< .001
***(B) Discontinuation of tenofovir due to toxicity***				
***Drug Class***				
PI/r	1.00		1.00	
NNRTI	2.04 (1.42–2.93)	<0.001	1.58 (0.93–2.70)	0.093
***Age*, *years***				
per 10 older	1.60 (1.34–1.90)	<0.001	1.43 (1.11–1.85)	0.005
***Calendar year***				
per more recent year	1.12 (1.03–1.22)	0.010	1.14 (1.00–1.31)	0.059
***Baseline eGFR*, *ml/min***				
per 10 lower	1.32 (1.21, 1.45)	<0.001	1.24 (1.08, 1.42)	0.002
***(C) Selective discontinuation of tenofovir***				
***Drug Class***				
PI/r	1.00		1.00	
NNRTI	3.93 (2.56, 6.05)	<0.001	2.77 (1.49, 5.12)	0.001

* adjusted for age, gender, black ethnicity, mode of HIV transmission, weight, hepatitis C co-infection status, AIDS diagnosis, baseline CD4+ count and nadir, viral load at cART initiation, year of starting cART, diabetes, use of blood pressure lowering drugs at baseline, baseline eGFR and stratified by clinical center.

eGFR was calculated using the chronic kidney disease epidemiology collaboration formula. [21]

List of abbreviations: cART, combination antiretroviral treatment; CI, confidence interval; eGFR, estimated glomerular filtration rate; NNRTI, non-nucleoside reverse transcriptase inhibitor; PI/r, ritonavir-boosted protease inhibitor; RH, relative hazard.

### Reasons for tenofovir discontinuation and risk of toxicity-driven discontinuation

**Fig 2:**
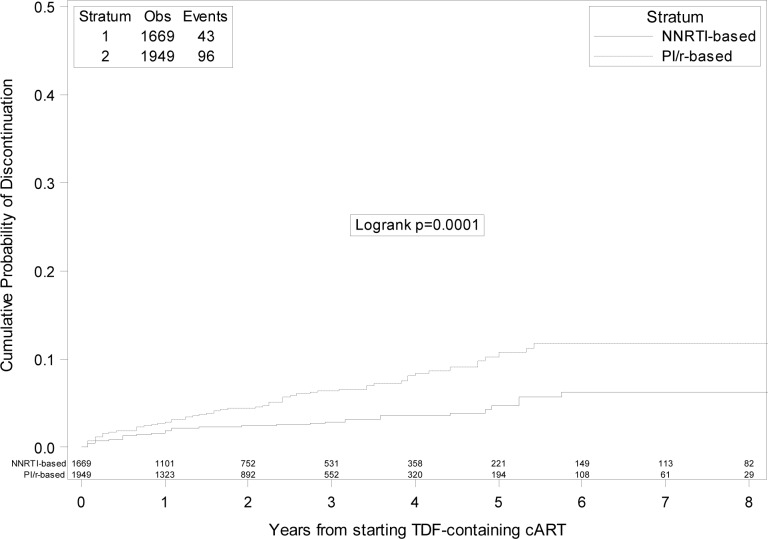
Kaplan-Meier estimates of risk of tenofovir discontinuation due to toxicity in boosted protease inhibitors versus non-nucleoside reverse transcriptase inhibitors groups.

A half (n = 139, 50.2%) of the discontinuations were driven by toxicity. Among these, 78/139 (56.1%) were motivated by renal toxicity (64 patients) or by osteopenia/osteoporosis (14 patients). Interruptions due to toxicity accounted for 31% (43/139) and 69% (96/139) of the discontinuations among patients who started PI/r and NNRTI, respectively. The other reasons for TDF discontinuation reported by the treating physician were non-adherence (7.6%%), simplification (15.5%), failure (11.2%), and other/unknown causes (11.2%). The 5- year KM estimates of TDF discontinuation due to toxicity were 10.7% (95%CI: 8.1–13.4) vs. 4.7% (95%CI: 2.9–6.5) for discontinuation due to toxicity in the PI/r and NNRTI group, respectively (Log-rank p = 0.0001) (Fig 2).

In a multivariable Cox regression analysis, initiating treatment with PI/r (compared with starting a NNRTI, HR 1.58; 95%CI 0.93–2.70), older age (per 10 years increase, HR 1.43; 95%CI 1.11–1.85) and lower baseline eGFR (per 10 ml/min decrease, HR 1.24; 95%CI 1.08–1.42) were independent predictors of TDF discontinuation due to toxicity (Table 3B). When discontinuation due to kidney toxicity was used as outcome measure of a separate Cox regression analysis, drug companion was not associated with the risk of TDF discontinuation (HR 1.31 of PI/r vs. NNRTI; 95%CI 0.58–2.98) and a lower baseline eGFR was the only independent predictor of TDF discontinuation (per 10 ml/min decrease, HR 1.46; 95%CI 1.21–1.76).

When a mixed linear model was used to estimate average eGFR trajectories, people who had started TDF co-administered with a PI/r, rather than a NNRTI, had a steeper eGFR reduction over time, but the difference was not statistically significant (mean change in eGFR per year in the PI/r group: -1.5; 95%CI [-1.9;-1.2] vs.–mean change in the NNRTI group: -1.2; 95%CI [-1.7;-0.70], p = 0.32).

### Selective tenofovir discontinuations

**Fig 3:**
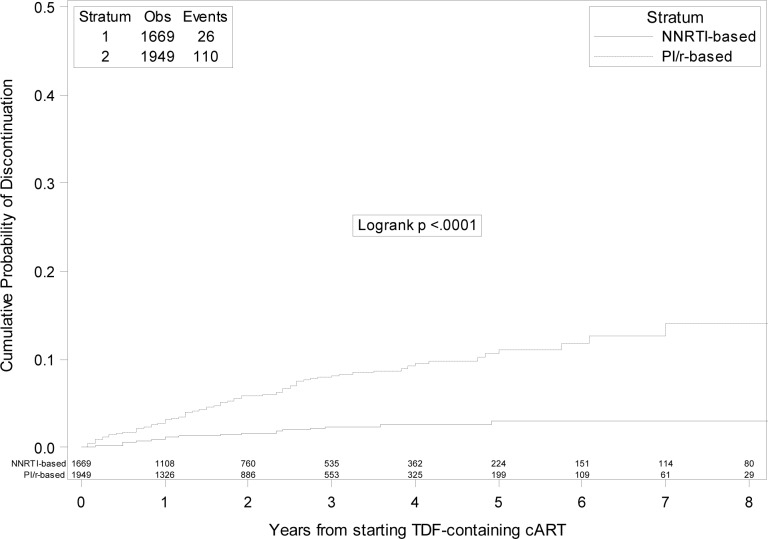
Kaplan-Meier estimates of risk of selective tenofovir discontinuation in boosted protease inhibitors versus non-nucleoside reverse transcriptase inhibitors groups.

One hundred and thirty-six selective TDF discontinuations were observed during the study follow-up. After selective TDF discontinuation, 75 (55.2%) patients switched to an abacavir/lamivudine-containing regimen, 34 (25%) to a regimen containing the sole lamivudine or emtricitabine, 8 (5.9%) to a zidovudine/lamivudine-containing regimen, 1 (0.7%) to other NRTI-combinations and 13 (9.6%) to NRTI-sparing combinations. A detailed description of the composition of the regimen started after the discontinuation according to the initial third drug is depicted in Table 4. The 5 year KM estimates in the PI/r vs. NNRTI group are illustrated in Fig 3. Using multivariable Cox regression analysis, initiation of PI/r (HR 2.77 vs. starting a NNRTI; 95%CI 1.49–5.12) was the only independent predictor of selective TDF discontinuation (Table 3C).

**Table 4:** Composition of the NRTI back-bone of the regimen started after the discontinuation of tenofovir (rows), according to the initial third drug (columns)–Selective tenofovir discontinuations.

	Regimen started					
New regimen after stop of TDF	TDF/FTC or TDF/3TC					
	plus					
	Efavirenz	Nevirapine	Lopinavir	Atazanavir	Darunavir	Any drug
NRTI-sparing regimen	0	0	5	2	6	13 (9.6%)
Abacavir/lamivudine	17	3	17	19	19	75 (55.1%)
Zidovudine/lamivudine	2	0	5	1	0	8 (5.9%)
Lamivudine or emtricitabine only	0	0	2	21	11	34 (25%)
Didanosine ± lamivudine or emtricitabine	4	0	1	0	0	5 (3.7%)
Other NRTI combinations	0	0	0	1	0	1 (0.7%)
Total	23 (16.9%)	3 (2.2%)	30 (22.1%)	44 (32.3%)	36 (26.5%)	136 (100%)

## Discussion

In our study a non-negligible proportion of naïve patients, initiating TDF as part of their first antiretroviral regimen started when they were ART-naive, underwent discontinuation of the drug. We found that the probability of TDF discontinuation by 2 years of treatment was 7.4% and, in 50% of our participants, the main reason leading to discontinuation was toxicity. By 5 years, 14.1% of patients had discontinued TDF. These rates were significantly higher than those reported in previous randomized controlled studies in patients using TDF, in which the proportion of toxicity events leading to discontinuation ranged from 0 to 3% by 2 years from starting the drug.[13–18, 22] There may be multiple reasons for this apparent discrepancy. Patients with pre-existing kidney disease or with risk factors for TDF-associated renal impairment, such as cardiovascular or metabolic diseases, concomitant nephrotoxic medications, low body weight, advanced age and lower CD4 cell count are typically under-represented in clinical trials.[22] There is an issue with this as patients in trials are different from those seen in clinical practice, who are aging populations, with advanced HIV infections and multiple comorbidities. Moreover, treating physicians could be more prone to modify treatment in the clinical setting than during trials. In clinical practice, there are less restriction to switching than in some of the trials and if there are prior beliefs among clinicians that a drug is likely to drive a specific toxicity the appearance of one of these may lead to early treatment interruptions or drug switches, even in presence of only mild, not clinically significant side effects, such as initial GFR reduction or bone mineral density loss. Similarly, unconventional less-drug regimens, such as NRTI-sparing or dual therapies with lamivudine, used in 23% and 18% of patients discontinuing TDF in our cohort, respectively, might have been pursued by clinicians in the attempt of both reducing costs and preventing long-term drug toxicities.

Nevertheless, our results prove that TDF was maintained as part of ART regimens in the majority of patients, for a period of observation of >5 years. Discontinuation rates of TDF appear to be still lower than those reported for other drugs. Previous observational studies reported rates of discontinuation after 2 year of more than 30%, for third-drugs [23] and for abacavir/lamivudine.[24] Consistently with these results, a study on determinants of modification of the first ART in a large cohort of European and North American patients suggested that TDF/emtricitabine had the lowest rate of switch/change and interruption, compared to zidovudine/lamivudine, abacavir/lamivudine and other backbone combinations.[25] All together, these findings support current guidelines indicating TDF as component of most suggested first-line ARV regimens, based on its high potency and safety profile.[26] In our study, co-administration with a PI/r was consistently associated with higher rates of TDF discontinuation, regardless of the chosen endpoint (although the difference was not statistically significant when the discontinuation due to renal toxicity was taken into account, mainly because of the lack of power of this secondary analysis). This finding seems to be supported by biological evidence. Previous studies have suggested that PI/r could slow TDF renal clearance and increase its plasma and renal tubular intracellular concentrations, via different mechanisms, including a blockage of the tubular renal transporter of TDF (the multidrug resistance associated proteins), P-glycoprotein activity inhibition and an increased intestinal absorption.[27, 28] As a possible consequence, higher rates of renal toxicity due to TDF, when associated with PI/r, have been shown in several studies.[4, 8, 9, 11] Of note, no significant differences were found when different PI/r were compared to each other. All together, these finding suggest that durability of TDF-containing treatment may depend on its companions and that use of PI/r should be evaluated with caution in patient with other risk factors for TDF premature discontinuation and/or toxicity.

On the other hand, when we specifically investigated creatinine, no significant differences in term of eGFR decline was found comparing patients treated with TDF in combination with PI/r or NNRTI. Of interest, the effect was in the expected direction with people who initiated a PI/r showing a higher initial level of eGFR and a steeper slope of decline over follow-up when compared to those starting a NNRTI. Moreover, when discontinuation due to renal toxicity alone was considered as the outcome, no difference in rate of discontinuation was found between PI/r and NNRTI recipients. These findings suggest that, in clinical practice, TDF discontinuations is driven by parameters other than eGFR alone. Indeed, renal function assessment is likely to include also other parameters (such as proteinuria or phosphate level), which were not available in our database. Moreover, other causes, related or not related with direct drug toxicity, can be more important in the decision of TDF discontinuation. Nevertheless, we cannot exclude that the observed increased risk in the PI/r group could be due to treating clinicians’ beliefs regarding the interaction between TDF and PI/r or other unmeasured confounding.

Not surprisingly, the risk of TDF discontinuation due to toxicity and renal toxicity was higher among patients with a lower eGFR at baseline, in accordance with the results of a previous study.[5] When other options are available, use of TDF should be avoided in those with compromised renal function before treatment initiation.

Our study has some limitations that merit to be acknowledged. First, as mentioned above, this is a comparison in the observational setting so that confounding is likely to be an issue. Second, the reasons for TDF discontinuation are those reported by the physician and therefore subjective by definition. Clinicians’ strategies may also vary by clinical sites (although Cox models were stratified by site). Moreover, only the main reason of discontinuation was taken into account, although only in seven cases a secondary reason for TDF discontinuation was reported, thus it is unlikely to have influenced our results. Third, there was a high percentage of unknown/other causes of discontinuation which is difficult to handle in the statistical analysis without making some strong assumptions. Fourth, as already mentioned, renal function was evaluated basing solely on eGFR and other markers of renal damage, such as urine dip stick analysis, phosphatemia or glycosuria, were not available. Fifth, due to the study time-frame, in this analysis we were not able to explore the rate of TDF discontinuation when it is prescribed in association with integrase inhibitors or coformualted with rilpivirine, because these regimens were introduced in more recent years.

In conclusion, our study had showed that frequency of TDF discontinuation in clinical practice is relatively low but much higher than that estimated in clinical trials. The co-administration of TDF with PI/r versus NNRTI and lower eGFR at initiation of the TDF-based regimen were independently associated with a higher risk of TDF discontinuation but discontinuation of TDF due to toxicity might be driven by parameters other than eGFR alone. Further studies are needed to clarify the possible interaction between TDF and the PI/r class which may lead to renal toxicities in patients with HIV treated with these drugs.

## Supporting Information

S1 File Plos_submission.xls. Data set
(XLS)
